# A review of patients who suddenly deteriorate in the presence of paramedics

**DOI:** 10.1186/1471-227X-8-9

**Published:** 2008-07-26

**Authors:** Malcolm J Boyle, Erin C Smith, Frank Archer

**Affiliations:** 1 Monash University, Department of Community Emergency Health and Paramedic Practice, P.O. Box 527, Frankston 3199, Victoria, Australia

## Abstract

**Background:**

The report of the Ministerial Review of Trauma and Emergency Services in Victoria, Australia, recommended that paramedics be permitted to divert to the closest hospital in incidences of life threatening situations prior to and during transport. An audit of patients that suddenly deteriorated in paramedic care was recommended by the Ministerial Review. The objective of the study was to identify the number and outcome of patients who suddenly deteriorated in the presence of paramedics.

**Methods:**

A retrospective cohort study of trauma patients who suddenly deteriorated in the presence of paramedics during 2002. As there was no standard definition, sudden deterioration was defined using a predetermined set of physiological criteria. Patient care record data of patients who suddenly deteriorated were compared with the State Trauma Registry to determine those who sustained hospital defined major trauma. Patient care records where hospital bypass was undertaken were identified and analysed. Ethics committee approval was obtained.

**Results:**

There were 2,893 patients that suddenly deteriorated according to predefined criteria. 2,687 (5.1% of the total trauma patients for 2002) were suitable for further analysis. The majority of patients had a sudden decrease in BP (n = 2,463) with 4.3% having hospital defined major trauma. For patients with a sudden decrease in conscious state or a total GCS score of less than 13 (n = 77), 37.7% had hospital defined major trauma; and a sudden increase/decrease in pulse rate and sudden decrease in BP (n = 65), 26.2% had hospital defined major trauma. Only 28 documented incidents of hospital bypass were identified.

**Conclusion:**

This study suggests that the incidents of patients suddenly deteriorating in the presence of paramedics are low and the incidence of hospital bypass is not well documented.

## Background

Evidence for the management of patients who suddenly deteriorate in the presence of a paramedic crew, either at the scene or during transport, is limited. There is no effective guidance as to which types of trauma patients are more likely to deteriorate suddenly and what the appropriate triage strategy is for those patients.

There were two significant pre-hospital care questions that remained unresolved following the report of the Ministerial Review of Trauma and Emergency Services in Victoria (MROTESV), Australia. They were, "is mechanism of injury a useful predictor in pre-hospital trauma triage?" and "what is the appropriate triage strategy for patients who severely deteriorate at the scene or during transport?".[1] Both of these unresolved questions are potentially controversial, impact significantly on EMS operations, and are crucial to the success of the state trauma system. Based on this report and these unresolved questions, a project was conducted to compile a complete state-wide dataset of trauma incidents attended by, and for patients transported by, the State's two Emergency Medical Services (EMS) to assist in answering the two questions.

The authors of the MROTESV report stated "The task force and working party gave close consideration to the most appropriate triage strategy for patients who severely deteriorate at the scene or during transport. The task force and working party agree that Ambulance and Mobile Intensive Care Ambulance (MICA) Paramedics must be allowed to default from hospital bypass in circumstances of an immediately life threatening situation during transport." The implication of this compromise position is demonstrated by the intention that the "audit of this patient group should be a priority for the state trauma committee to enable future change in triage guidelines, if appropriate, and to support education strategies and foster compliance with recommended triage guidelines, especially for ASV." [1]

Current EMS procedures dictate that a lower level hospital is bypassed for a trauma centre, however, for a patient that is suddenly deteriorating the destination of the patient, to some extent, still remains at the discretion of the attending paramedics, especially in rural areas. The objective of the study was to identify the number and outcome of patients who suddenly deteriorated in the presence of paramedics.

## Methods

The study was a retrospective cohort study of trauma patients who suddenly deteriorated and were attended to, and or transported by, Victorian EMS during 2002.

The study was conducted in Victoria, a south eastern state of Australia. Victoria covers approximately 227,590 square kilometres with a population of approximately 4.9 million people during the study period (49% males and 51% females). [2]

The Metropolitan Ambulance Service (MAS) provides the EMS service for the greater Melbourne metropolitan area which covers roughly 7,694 square kilometres and a population of some 3.5 million people at the time of the study. [3] Rural Ambulance Victoria (RAV) services the remaining 1.4 million people covering roughly 219,896 square kilometres of Victoria. [4]

The state of Victoria has a two tier EMS response. The first tier is the Ambulance Paramedic who has core Advanced Life Support (ALS) skills. The second tier is the Mobile Intensive Care Ambulance (MICA) Paramedic who has a broader range of ALS skills including intubation and a greater range of drugs.

No electronic EMS clinical data repository was available in Victoria at the time of data collection. Consequently, each EMS Patient Care Record (PCR) for 2002 was manually reviewed. All trauma PCRs were retrieved, and then each individual trauma incident was analysed to establish eligibility for inclusion into the study. Eligibility was determined using pre-set inclusion and exclusion criteria.

Trauma patients transported by emergency ambulances in Victoria from the 1^st ^January 2002 to the 31^st ^December 2002 were eligible for inclusion in the study.

The sudden deterioration data repository included all patients who had sustained trauma through a road traffic accident, industrial incident, burns/explosions, or other trauma not specifically listed here, were transported by ambulance, and who suddenly deteriorated in the presence of paramedics either at the scene or enroute to a medical/hospital facility.

Patients were excluded from the sudden deterioration data repository and further analysis, if the patient was transported as a result of an inter-hospital transfer, the PCR had insufficient information to determine if the patient had sustained trauma, what type of trauma, and insufficient information to determine if the patient deteriorated suddenly, or a patient who did not suddenly deteriorate, either at the scene or enroute to hospital.

Each PCR was reviewed by a researcher to establish eligibility as predetermined by the inclusion and exclusion criteria. The PCRs that met the inclusion criteria had specific data entered into a secure relational database (Microsoft Access™ Version 10 SR2, Redmond, Washington, U.S.A.) written specifically for the project. The dataset was then reviewed to ensure that only one PCR per patient per incident was in the data repository.

We defined sudden deterioration as, "A person's condition is said to have suddenly deteriorated if there is a decrease in any of the physiological status components from the last recorded observations to the most recent. This deterioration is in light of on-going management of the patient's overall condition. This time frame between the observations would normally be about fifteen minutes".

The sudden deterioration criteria were:

• Change in pulse rate: either, a sudden increase in pulse rate of 20 beats per minute above the previous reading, or greater than 110 beats per minute, or less than 50 beats per minute.

• Change in blood pressure: either, a sudden drop of blood pressure of 20 mmHg or more since the last reading, or a fall below 90 mmHg systolic.

• Change in respiratory rate: either, a sudden increase in respiratory rate of 10 breaths per minute above previous reading, or greater than 29 breaths per minute or less than 10 per breaths minute.

• Change in conscious state: either, a sudden decrease in conscious state of 2 points in either component (eye opening, best verbal response, best motor response) of the Glasgow Coma Scale (GCS), or a total score of less than 13.

• New uncontrollable haemorrhage.

• Cardio-respiratory arrest.

Descriptive data analysis was undertaken using SPSS (Statistical Package for the Social Sciences Version 14.0, SPSS Inc., Chicago, Illinois, U.S.A.). Descriptive statistics were used to summarise the demographic data. All confidence intervals (CI) are 95%.

Ethics approval for the project was obtained from the Monash University Standing Committee for Ethics in Research on Humans and the Victorian Department of Human Services Ethics Committee.

## Results

There were 2,893 patients who met the criteria for sudden deterioration, of these, 206 incidents had insufficient injury type data leaving 2,687 for further analysis. The 2,687 patients amounted to 5.1% of the total number of trauma patients transported or seen by the EMS (n = 53,039) for 2002.

Of the patients who were deemed to have deteriorated suddenly, MAS attended to approximately 76% of this group whilst RAV attended to approximately 24%. This ratio is similar to that for all trauma incidents attended by each service for 2002. [5]

The gender distribution was 64% males and 36% females. Mean age was 39.6 years, standard deviation of 19.57 years. Median age was 36 years, age range was 1 month to 97 years. The age information is based on the actual age when there was a date of birth to calculate the age or an approximate age when there was no date of birth.

Of the patients 4.3% were paediatric (< 15 years), 23.1% of patients were elderly (> 55 years), 72.2% were in the adult group, the remainder had no age listed on the PCR.

The clinical profile of patients who suddenly deteriorated can be seen in Table 1.

**Table 1:** Sudden Deterioration Types

**Deterioration Type**	**N** **(2893)**	**% of Total ** **Sudden ** **Deterioration**
Sudden Increase/Decrease in Pulse Rate	174	6.0
Sudden Decrease in Blood Pressure (> 20 mmHg), or < 90 mmHg	2463	85.1
Sudden Increase/Decrease in Respiratory Rate	36	1.2
Sudden Decrease in Conscious State or a Total Score of Less Than 13	77	2.7
New Uncontrollable Haemorrhage	0	0
Cardio-Respiratory Arrest	20	0.7
Sudden Increase/Decrease in Pulse Rate and Sudden Decrease in Blood Pressure	65	2.2
Sudden Decrease in Blood Pressure and Sudden Decrease in Conscious State	29	1.0
Sudden Increase/Decrease in Pulse Rate and Sudden Decrease in Blood Pressure and Sudden Decrease in Conscious State	10	0.3
Sudden Increase in Pulse Rate and Respiratory Rate	7	0.2
Sudden Increase/Decrease in Pulse Rate and Sudden Decrease in Conscious State	11	0.4
Sudden Increase/Decrease in Pulse and Respiratory Rate and Sudden Decrease in Blood Pressure	1	0.03

The ISS ranged between 4 and 75, mean was 27.1, standard deviation of 13.49, and median ISS of 25. The ISS was missing for five patients with hospital defined major trauma (Table 2). For the patients who died the greater percentage had an ISS of 25 to 26, the remaining deaths were distributed amongst the higher ISS scores.

**Table 2:** Hospital Defined Major Trauma [1]


Major Trauma Criteria

Death after injury

Admission to an Intensive Care Unit for more than 24 hours, requiring mechanical ventilation

Urgent surgery for intracranial, intra-thoracic, or intra-abdominal injury, or for fixation of pelvic or spinal fractures

Injury Severity Score (ISS) > 15

Serious injury to two or more body systems (excluding integumentary)

**Figure 1:**
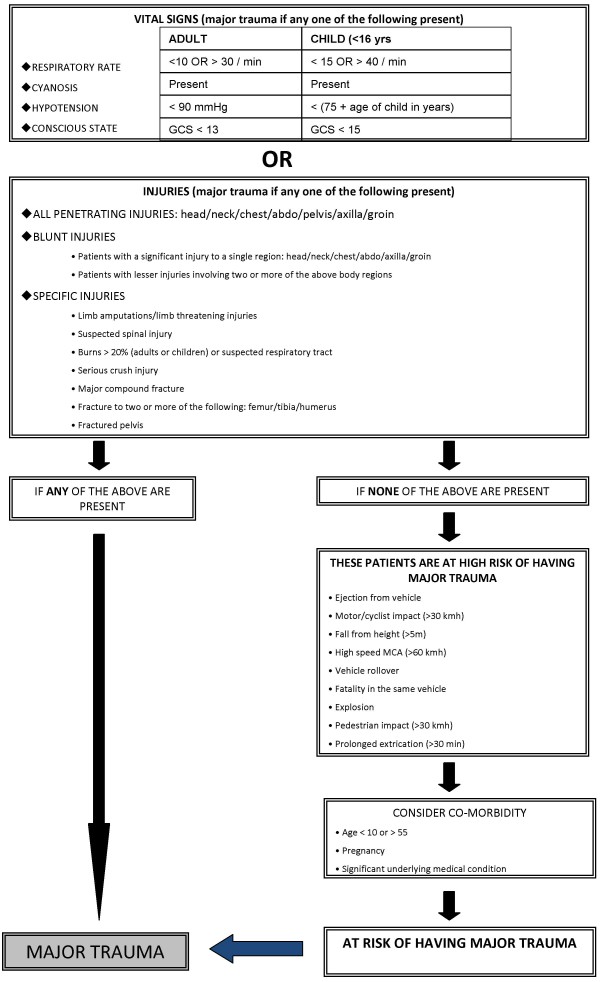
ROTESV Prehospital Major Trauma Criteria [1]. Copyright: Prehospital and Disaster Medicine.

**Figure 2:**
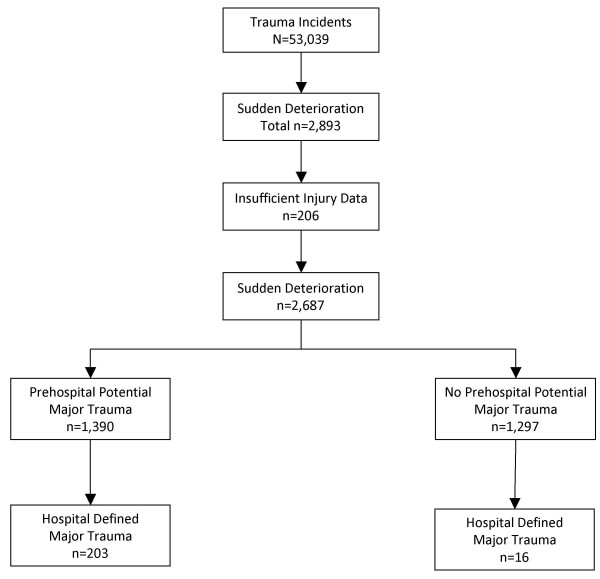
Total Trauma Incidents, Sudden Deterioration, and Prehospital Potential Major Trauma with Hospital Defined Major Trauma.

Of the patients who suddenly deteriorated (n = 2,687), 51.7% (n = 1,390) had prehospital potential major trauma (Figure 1), 7.6% (n = 203) had hospital defined major trauma, and 2% (n = 54) died. For the patients with prehospital potential major trauma (n = 1,390), 14.6% (n = 203) went on to have hospital defined major trauma with 26.6% (n = 54) of this group subsequently dying. See Figure 2 for the distribution.

### Analgesia administration and sudden deterioration

Early in the data collection stage it was noted that a number of patients who met the sudden deterioration criteria also had severe pain, greater than 7 to 8 out of 10, and a number also had a sudden decrease in blood pressure following analgesia administration.

Of the 2,568 patients who had a sudden fall in blood pressure (> 20mmHg, or < 90 mmHg), 827 (32.2%) had also received analgesia, either morphine sulphate 25.7% (n = 213), methoxyflurane (Penthrane) 63.7% (n = 527), or both Penthrane and morphine sulphate 10.5% (n = 87). Of the sudden fall in blood pressure group 0.1% was associated with other drugs, namely:

• following sedation to achieve intubation (n = 13)

• following sedation to maintain intubation (n = 41)

• following midazolam to control seizure activity (n = 2)

This finding will require further analysis and interpretation with is outside the scope of this paper.

### Trauma types

**Figure 3:**
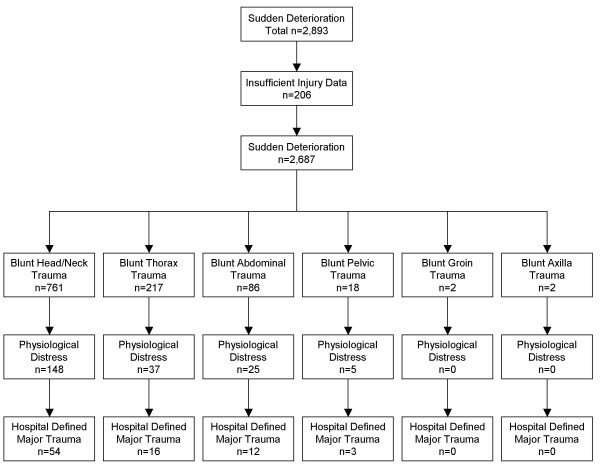
Sudden Deterioration, Blunt Trauma, Physiologically Distress and Hospital Defined Major Trauma.

There were 129 patients identified who received significant blunt trauma leading to prehospital potential major trauma who subsequently had hospital defined major trauma. The main body region was the head/neck (n = 76) followed by the thorax (n = 35), the abdomen (n = 14) and pelvis (n = 4). From this cohort 35 patients died, the main cause of death was significant blunt trauma to head/neck (n = 19) followed by the thorax (n = 5), and the abdomen (n = 11). See Figure 3.

**Figure 4:**
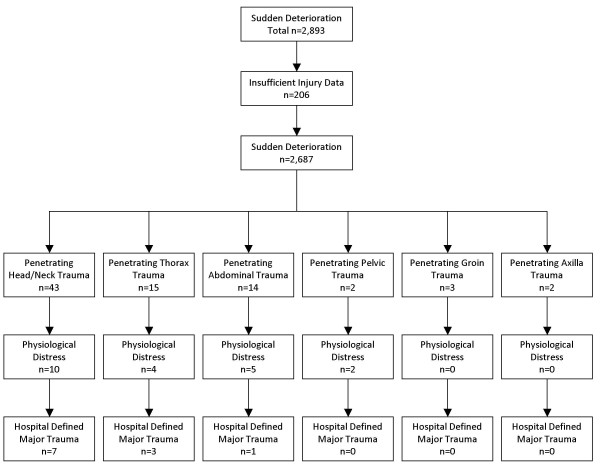
Sudden Deterioration, Penetrating Trauma, Physiologically Distress and Hospital Defined Major Trauma.

There were 21 patients identified who received some form of penetrating trauma leading to prehospital potential major trauma who subsequently had hospital defined major trauma. The main body region was the head/neck (n = 12) followed by the abdomen (n = 5), the thorax (n = 2), and groin (n = 2). From this cohort 12 patients died, the main cause of death was penetrating trauma to the head/neck (n = 8) followed by the thorax (n = 2), and the abdomen (n = 2). See Figure 4.

### Time intervals

The scene time for patients who suddenly deteriorated was available for 92% (n = 2,666) of incidents, mean scene time was 18.5 minutes (range 0 minutes to 262 minutes, CI 18.01 to 19.06), median scene time was 15 minutes. The scene time is measured from the time the paramedics arrive at the patient until they depart the incident location for the receiving facility. The scene time for non trapped patients was available for 92% (n = 2,618) of the incidents, mean scene time was 17.9 minutes (range 0 minutes to 170 minutes, CI 17.45 to 18.38), median scene time was 15 minutes. The scene time for trapped patients was available for 84% (n = 48) of the incidents, mean scene time was 52.5 minutes (range 7 minutes to 262 minutes, CI 41.13 to 63.96), median scene time was 43.5 minutes.

The transport time for patients who suddenly deteriorated was available for 95% (n = 2,754) of incidents, mean transport time was 18.9 minutes (range 0 minutes to 160 minutes, CI 18.33 to 19.4), median transport time was 15 minutes. The transport time is measured from the time the paramedics depart the incident location for the receiving facility until they arrive at the receiving facility.

If we use the call received time as a surrogate for the time of the trauma incident then the number of incidents available for the time of incident to hospital analysis is 81% (n = 2,342). The mean time from the incident to arrival at hospital was 50.8 minutes (range 7 minutes to 424 minutes, CI 49.69 to 51.88), median time from the incident to arrival at hospital time was 45 minutes. The time from the incident to arrival at hospital is measured from the time the emergency call is received by the communication centre until they arrive at the receiving facility.

The number of incidents for each time group varied as some of the required times were often missing from the PCR or were inaccurate (non-sequential).

### Patient destinations

For those trauma patients with hospital defined major trauma (n = 203), 66% (n = 134) were transported directly to one of the state Major Trauma Service (MTS) hospitals situated in Melbourne. There were 76.9% of this cohort who had a transport time to hospital less than 30 minutes, 17.1% who had a transport time to hospital between 30 and 60 minutes, and 6% who had a transport time to hospital greater than 60 minutes.

There were 13.3% (n = 27) of trauma patients who were transported to one of the nine Regional Trauma Service (RTS) hospitals. The remaining patients were transported to a Primary Injury Service (PIS) hospital.

### Hospital bypass/diversion

We identified 117 incidents where the ambulance crew bypassed their intended hospital destination to another within the state hospital system. Of these, 28 had the reason for bypass documented with all bypasses due to the intended emergency department being on ambulance bypass as a result of overcrowding. The majority (n = 21) of hospitals were metropolitan based, with six being in the country, and one undefined hospital. For the remaining 89 incidences, we were not able to determine the reason for bypass.

There was no documented incidence where an ambulance diverted to a closer lower level trauma facility from their intended high level trauma facility due to the patient's condition suddenly deteriorating.

## Discussion

This study is the first in Victoria and Australia, and possibly internationally, to use a complete state EMS dataset, and not data from a state or hospital trauma registry, to investigate the characteristics of patients who suddenly deteriorate in the presence of paramedics.

There is a lack of literature describing the actions of paramedics in response to a patient suddenly deteriorating in their care and what actions were taken, including hospital bypass.

Several studies have demonstrated that patient outcomes were improved when trauma patients were taken to a level 1 trauma centre. [6-10] Prior to setting up the Victorian trauma system an analysis was undertaken by Cooper et al into preventable deaths and management errors in Victorian hospitals. The study found that the major metropolitan hospitals with higher trauma admissions (later designated as level 1 trauma centres) had significantly less management errors and preventable deaths compared to other hospitals with lower trauma admissions. [6] Studies undertaken in the USA demonstrated that trauma patients had better outcomes when managed in a level 1 trauma centre compared to lower level trauma facilities. [8-10] A study conducted in the United Kingdom by Freeman et al showed that only patients with multiple trauma and head injury had better outcomes in a high volume trauma centre that did other patients. [7]

A study by Macken et al in south-eastern Sydney, New South Wales, Australia, identified an overtriage rate of approximately 81% for hospital bypass using similar prehospital triage criteria to that used in this study. Following bypass of a lower level trauma facility to the level 1 trauma centre 34% of trauma patients involved in hospital bypass were discharged from the emergency department. [11] In a Victorian prehospital trauma study by Boyle et al looking at prehospital potential major trauma, an overtriage rate of approximately 90% was identified. [5]

Of the patients who suddenly deteriorated, 85% had a sudden decrease in blood pressure greater than 20 mmHg, or a blood pressure less than 90 mmHg, in the initial or subsequent vital sign sets. This observation should be interpreted with some caution for two reasons. First, the underlying pathology caused by the trauma is highly likely to be a confounder to this result. Second, the definition of "Sudden Decrease in Blood Pressure" included a relative change of 20 mmHg, or greater, which may have been too sensitive. Of the 2,463 patients in this category, only 13.2% (n = 70) could be clearly identified with an initial blood pressure less than 90 mmHg. However, the inclusion of the relative change has provided some interesting insights. The observations from this analysis of patients who suddenly deteriorated were unexpected. The number is clearly related to the proactive criteria we chose to identify sudden deterioration, particularly the use of "a change factor" rather than absolute numbers. In particular, the use of a change of 20 mmHg in blood pressure. However it is reasonable, clinically, to regard a sudden drop of this nature over a 15 minute interval as being clinically significant. We were unable to identify any literature for patients who suddenly deteriorate that describe a measurable "change factor", like a drop of blood pressure of 20 mmHg.

It is possible that the fall in blood pressure in some patients administered analgesia was a reduction from an artificial elevation as a consequence of pain or other sympathetic stimuli, in which case the fall may well have been clinically reasonable, particularly if the blood pressure remained over 90 mmHg systolic. It is possible in some patients that the fall in blood pressure was as a consequence of analgesia and may have been dose-related. A study by Semenkovich and Jaffe, even though in cardiac patients, demonstrated that 2.2% of patients with pain and no myocardial infarction had hypotension following morphine sulphate administration. [12] It is also feasible that the fall in blood pressure was a direct consequence of the trauma pathology unfolding in some patients. This observation of a change in blood pressure requires further analysis and understanding, especially in head injury patients and in the use of analgesia in prehospital trauma care. It is also difficult to comment on the potential affects of interactions between the patient's current medication and the use of morphine sulphate and Penthrane for pain management. The patient's medication list was not always included on the PCR, and if it was, the dosage of the patient's drugs was often missing.

If the "sudden decrease in blood pressure (> 20 mmHg, or < 90 mmHg)" patients are excluded because of the potentially oversensitive nature of the criteria, only 430 (15%) patients deteriorated. A potentially more realistic number.

We have analysed the scene time, transport time and total incident time to ascertain if EMS crews were spending too long at the scene, transporting the patient to distant hospitals, and whether the patient was in a receiving facility within as short a timeframe as possible, given the patient's deteriorating condition,

We found the average scene time in an urban area of 16.5 minutes compares to the study by Carr et al of 13.5 minutes and 13.4 minutes in the study by Al-Ghamdi. [13,14] When comparing rural average scene times we found the time by Carr et al was considerably lower, 15.06 minutes to 26.8 minutes in this study. [14] The study by Goodacre et al in the West Yorkshire region of England found the average scene time for non-trapped trauma patients was 26 minutes, which was greater than in this study, 17.9 minutes. [15] The combining of urban and rural scene times appears to artificially lower the overall scene time. Rural scene times are often affected by distances, a lack of resources, or a delay in resources arriving, e.g. road accident rescues crews, as they are predominately volunteers, especially in this state.

The average transport times reported by Al-Ghamdi, 9.8 minutes, and Carr et al, 10.78 for urban and 17.37 for rural, were less than in this study, 17.2 minutes for urban and 24.6 minutes for rural. [13,14] An issue not often identified in the international studies is the location of hospitals within the study geographic area and the distribution of EMS units. In urban Victorian the average response time and transport time vary by several minutes, highlighting that ambulances may have further to travel to hospital, especially if the hospital is on bypass. In rural Victoria the average response times and transport times are similar as the town contains an EMS station and hospital.

The total incident time demonstrates that on average the trauma patient was in a hospital facility within an hour of the incident, with the majority of these being a MTS or RTS. When comparing the results from this study, for urban EMS, to two studies from the USA there was a considerable time difference between this study, 47.5 minutes, compared to a study by Feero et al of 29.3 minutes for patients with an unexpected death and 30.96 minutes in a study by Carr et al. [16,14] However, this study covered a larger area and greater population which may account for the large variation. The study by Al-Ghamdi found that the average total incident time for traffic accidents was 34.6 minutes. [13] Carr et al found the average total incident time for rural ambulances was 43.17 minutes compared to this study where it was 80.3 minutes. [14] It would appear that the greater the geographical area covered by the EMS the greater the total incident time, this includes primary response helicopter trips from rural locations to a MTS.

Even though there are demonstrated benefits from transporting trauma patients to a level 1 trauma centre it would appear that patients with multiple trauma and head injuries receive the most benefit. In this study it could be argued that patients who dropped their BP by 20 mmHg or more following pain relief and remain stable following this initial drop in BP, and do not have multiple trauma or a head injury, do not need to be transported to a level 1 trauma centre. The remaining patients who met the sudden deterioration criteria should have been transported to a level 1 trauma centre or the highest level trauma facility available.

The trauma patients who need to be diverted to the closest trauma facility would be those who, despite ongoing appropriate management, are failing to have their physiological status returned to within normal limits.

This study was designed to identify those situations where the patient suddenly deteriorated and the paramedic crew diverted to a hospital other than a MTS for initial resuscitation. Without knowing the hospital bypass status at the time and the lack of documentation about hospital bypass on the PCR, it is difficult to comment on the bypass strategy. We have identified some Metropolitan Trauma Service (MeTS) hospitals that were bypassed for another MeTS. There were also Primary Injury Service (PIS) hospitals (major metropolitan private hospitals) that received trauma patients after bypassing a MeTS hospital. However, none of these patients transported to the PIS had hospital defined major trauma, but they did have prehospital potential major trauma. The issue of hospital bypass and diversion requires additional research to gain an accurate picture of the bypass and diversion numbers and reasons.

This study has identified that there are very few trauma patients who suddenly deteriorate in the presence of paramedics. Given the proven benefit of major trauma patients being managed in a MTS and that we have no evidence these patients are being diverted to lower level trauma facilities for resuscitation, it would be reasonable to change the triage strategy for these patients and extend the current transport time to a MTS from 30 to 60 minutes. Following this change a prospective audit would be needed to monitor the affect of the change. Paramedics will continue to divert to a closer hospital if they are not confident in managing the patient's deteriorating condition. Rural paramedics are less likely to transport a patient, no matter what their condition, to a hospital in a neighbouring town that is some distance away as they may not be confident in managing the patient or they do not want to "leave their town" without paramedic cover.

This study is subject to a number of limitations. Firstly, the data used in this study is based on analyses of PCRs identified during a review of all PCRS for the 12 month period. While the authors believe that all available trauma PCRs were included in the study, the possibility of missing PCRs cannot be discounted. Secondly, the lack of documentation on the PCR by paramedics regarding hospital bypass is not a true reflection of the actual occurrence of hospital bypass. Finally, as there was some required data missing from the PCRs, e.g. incident number, patient gender and age, we cannot be entirely sure that all ambulance data and state trauma registry data were linked successfully. Despite these limitations, this study has provided a unique insight into the trauma incidents attended by EMS crews in Victoria.

## Conclusion

This study suggests that the incidents of patients suddenly deteriorating in the presence of paramedics are low and that the phenomenon of a sudden decrease in blood pressure following analgesia for severe pain requires further investigation. The incidence of hospital bypass and diversion is not well documented and requires further attention by both state EMS. These results add to the knowledge base of trauma presentation in the pre-hospital setting, especially in Australia, and are the baseline for further studies.

## Abbreviations

ASV: Ambulance Service Victoria

## Competing interests

The authors declare that they have no competing interests.

## Authors' contributions

MJB and FA, devised the methodology for the project, ECS and MJB undertook the data analysis, all authors contributed to writing the manuscript. All authors read and approved the final manuscript.

## Pre-publication history

The pre-publication history for this paper can be accessed here:


